# Flexible and Washable Poly(3,4-ethylenedioxythiophene): Polystyrene Sulfonate/Polyvinyl Alcohol Fabric Dry Electrode for Long-Term Electroencephalography Signals Measurement

**DOI:** 10.3390/polym17050683

**Published:** 2025-03-04

**Authors:** Fangmeng Zeng, Guanghua Wang, Chenyi Sun, Jiayi Gao, Shanqun Ji, Quanxi Zhang

**Affiliations:** 1 School of Environmental & Resource Sciences, Shanxi University, Taiyuan 030006, China; 2 College of Textile Science and Engineering (International Institute of Silk), Zhejiang Sci-Tech University, Hangzhou 310018, China; 3 Zhejiang Yuntai Textile Co., Ltd., Quzhou 324200, China

**Keywords:** EEG signals, dry electrode, PEDOT: PSS, conductive fabric, smart textiles

## Abstract

Recent advancements in smart textiles have facilitated their extensive application in wearable health monitoring, particularly in brain activity measurement. This study introduces a flexible and washable fabric dry electroencephalography (EEG) electrode designed for brain activity measurement. The fabric dry electrode is constructed from electrically conductive polyester fabric with a resistivity of 0.09 Ω·cm, achieved by applying a PEDOT: PSS/PVA conductive paste coating on the textile substrate. A comparative analysis of the tensile properties between the conductive and untreated polyester fabric was conducted. The SEM images demonstrated that the PEDOT: PSS/PVA conductive polymer composite resulted in a uniform coating on the fabric surface. When enveloped in elastic foam, the fabric dry electrode maintained a low and stable electrode–skin contact impedance during prolonged EEG monitoring. Additionally, the short circuit noise level of the fabric dry electrode exhibited superior performance compared to both Ag/AgCl wet and finger dry electrode. The EEG signals acquired from the fabric dry electrode were comparable to those recorded by the Ag/AgCl wet electrode. Moreover, the fabric electrode effectively captured clear and reliable EEG signals, even after undergoing 10 washing cycles. The fabric dry electrode indicates good sweat resistance and biocompatibility during prolonged monitoring.

## 1. Introduction

The brain is recognized as the command center of the human body and stands as the largest and most intricate organ, comprising over 100 billion neurons [1]. Electroencephalography (EEG) represents the electrical activity generated by neuronal interactions within the brain, often referred to as the “window of brain function”. The EEG technique is the focus of significant research and possesses practical applications due to its high value. The EEG technique is widely used in brain research and has significant practical value. Compared to alternative brain activity measurement techniques, like functional magnetic resonance imaging (fMRI) and magnetoencephalography (MEG), EEG is distinguished by its superior temporal resolution and advantages related to convenience and cost-effectiveness. Consequently, EEG has become a widely utilized tool in various research domains, including the diagnosis of neurological disorders [2,3,4], emotion recognition [5], and brain–computer interaction [6]. EEG acquisition is facilitated by placing electrodes on the scalp, which measure the electrical potential signals generated by neuronal activity; these signals are subsequently amplified and recorded. Developing technology for acquiring EEGs with high-quality signals is critical for effectively monitoring brain activity.

Generally, EEG electrodes are classified into two categories: wet and dry. The conventional wet electrode, specifically the Ag/AgCl electrode, utilizes a conductive adhesive to enhance the electrode–scalp interface by reducing the contact impedance value to a range of 5–20 kΩ [7]. This configuration is regarded as the “gold standard” for EEG acquisition due to its efficacy in obtaining precise EEG recordings. Nevertheless, the application of a conductive adhesive presents several challenges, such as [8,9]: (1) the preparatory process before experimentation is complicated, time-consuming, and laborious; (2) the signal quality is compromised if the conductive adhesive becomes dehydrated and rigid over prolonged periods; (3) the use of a conductive adhesive can result in degradation of the subjects’ hair, leading to untidiness and potential irritation, thus negatively impacting user experience; and (4) these inherent limitations hinder the broader application of wet electrodes in routine scenarios outside of controlled laboratory or clinical environments.

Extensive advancements in dry electrode technology have been made to mitigate the issues associated with conductive gel, which offers practical usability and ease of operation. Generally, dry electrodes are designed in various configurations, including needle, comb, and column types, to facilitate penetration through hair and establish close contact with the scalp [10]. These dry electrodes usually utilize metals known for superior electrical conductivity, such as silver [11] or titanium [12]. However, they may induce discomfort due to the inherent rigidity of metal. The contact impedance increases because they make direct contact with the scalp, relying on small local amounts of sweat and water as the medium for ion exchange [13]. The microneedle electrode [14] has been developed to address the issue of high contact impedance. Nevertheless, it requires insertion into the scalp, thereby introducing a potential risk of infection and discomfort.

Textiles are the optimal substrates of dry electrodes because of their softness, flexibility, and comfort when wearing. The combination of textiles and electronics formed a new class of flexible and wearable smart textiles, enabling wide applications in wearable biopotential signals monitoring. Typically, textile-based EEG electrodes have been innovatively developed. Muthukumar et al. [15] designed a textile electrode for EEG measurement using copper-plated polyester fabrics. Muthukumar et al. [16] prepared a polyaniline-coated fabric–foam electrode to collect EEG signals. However, such textile-based EEG electrodes often exhibit high contact impedances like general dry electrodes. Poly(3,4-ethylenedioxythiophene): polystyrene sulfonate (PEDOT: PSS) demonstrates significant potential in reducing contact impedance through its ion–electron conductivity [17,18,19], as well as its good biocompatibility and stability. Tseghai et al. [20] developed EEG electrodes made from PEDOT: PSS/PDMS-printed cotton fabric, achieving low contact impedance and acquiring comparable signals as commercial electrodes. However, it is limited in collecting EEG signals in hairy areas.

This study developed a dry EEG electrode using PEDOT: PSS/PVA coated conductive fabric with flexibility, low contact impedance, and washability. The conductive fabric demonstrates excellent electrical conductivity while maintaining its textile properties, which can enhance user comfort with the fabric dry EEG electrode. The ion–electron conductivity of PEDOT: PSS could help achieve low contact impedance. The EEG signals collected by the fabric dry electrode are comparable to those of Ag/AgCl wet electrodes, even after 10 washing cycles. Moreover, not only can it be used at the forehead, but it can also be used at hairy sites for EEG signal measurement.

## 2. Materials and Methods

### 2.1. Materials and Chemicals

A 105 g per square meter (GSM) woven polyester fabric sourced from Hebei Hongda Weaving Factory, Shijiazhuang, China, was selected as the textile substrate for this study. To develop a high-conductivity polymer composite, PEDOT: PSS (1.5%, pH 2–2.5), procured from Shanghai Ouyi Organic Optoelectronic Materials Co., Ltd., Shanghai, China, was utilized in conjunction with ethylene glycol (EG, ≥99.5%), obtained from Zhejiang Tengyu New Material Technology Co., Ltd., Huzhou, China. Furthermore, polyvinyl alcohol (PVA, 1788), acquired from Wuxi Yatai United Chemical Co., Ltd., Wuxi, China, served as a fixing agent to enhance the tensile properties and speed of washing.

### 2.2. Conductive Fabric Preparation

**Figure 1:**
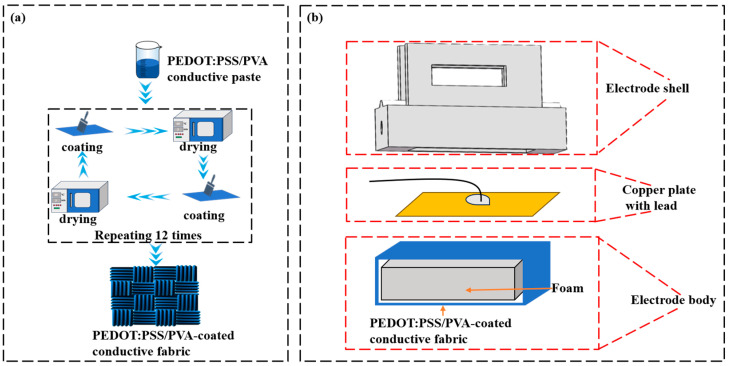

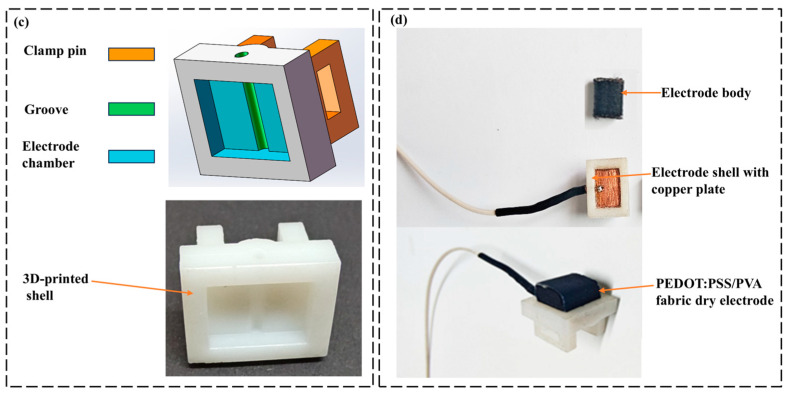
(**a**) Schematic illustration of the preparation process for conductive fabric; (**b**) structural design of the PEDOT: PSS/PVA fabric dry electrode; (**c**) model design of the electrode shell and its 3D-printed shell; (**d**) the assembled electrode and its components.

Firstly, the polyester fabric was immersed in deionized water and subjected to ultrasonic cleaning procedures to ensure the acquisition of a pristine fabric sample. Subsequently, 2.09 g of PVA powder was incorporated into 50 mL of water and stirred continuously at 98 °C utilizing a DF-101S magnetic stirrer (obtained from Shanghai Yushen Instrument Co., Ltd, Shanghai, China), which yielded a PVA solution with a mass fraction of 4%. A mixture comprising a 1:9 ratio of EG to PEDOT: PSS was prepared and stirred at ambient temperature for 30 min using the same DF-101S magnetic stirrer to achieve a homogeneous PEDOT: PSS-EG mixture. EG aims to enhance the electrical conductivity of PEDOT: PSS by physically increasing the carrier concentration or mobility [21]. An additional 11% of the weight of the previously prepared PVA solution (4% mass fraction) was then introduced to the PEDOT: PSS-EG mixture, followed by stirring at room temperature for another 30 min to produce the conductive paste. The PEDOT: PSS/PVA paste was then uniformly applied onto the cleaned polyester fabric. The coated fabric was subjected to a drying process at 80 °C for 5 min. This coating and drying procedure was repeated 12 times to produce PEDOT: PSS/PVA-coated conductive fabric with enhanced uniformity, in accordance with prior research findings [22]. Figure 1a presents a schematic illustration of the prepared conductive fabric.

### 2.3. Conductive Fabric Characterization

The increase in thickness should be assessed after coating. The thickness of the polyester fabric was measured both before and after coating, according to ISO 5084:1996(E) [23]. The surface micro-topography of the fabric before and after coating was examined utilizing an Ultra 55 FESEM (obtained from Carl Zeiss AG, Oberkochen, Germany) at an accelerating voltage of 3 kV. A gold layer was deposited on the unprocessed fabric sample to facilitate this analysis. The molecular structure of the PEDOT: PSS/PVA conductive polymer composite adhered to the fabric was characterized using a Nicolet 5700 FTIR (obtained from Thermo Fisher Scientific, Madison, WI, USA). The tensile properties of the coated fabric were evaluated using a YG026T-II (obtained from Ningbo Instrument & Textile Factory, Ningbo, China) following the guidelines established in ISO 13934-1 [24].

Additionally, the electrical characteristics, particularly the resistance of the conductive fabric designed for EEG electrode preparation, were assessed with a DEM11 digital multimeter (obtained from China Delixi Electric Sales Center, Hefei, China). The measurements were executed at four distinct locations within a 3 cm × 3 cm area of the conductive fabric (specifically, top left, top right, bottom left, and bottom right), and an average value was calculated to represent the overall resistance of the conductive fabric. The resistivity was subsequently determined using Equation (1), which is as follows:

(1) $$\rho =R\;\times \;\frac{S}{L}$$

where ρ = resistivity (Ω·cm), R = resistance (Ω), S = cross-section area (cm^2^), and L = length (cm).

The electrical characteristics of the conductive fabric were evaluated after washing, and the washing was carried out in accordance with the guidelines in GB/T8629-2017 [25]. The PEDOT: PSS/PVA fabric dry electrode underwent 10 washing cycles following the same standards. The impact of these washing procedures on the EEG signal quality of the fabric dry electrode has been systematically investigated.

### 2.4. PEDOT: PSS/PVA Fabric Dry Electrode Design

The EEG electrode was developed utilizing PEDOT: PSS/PVA-coated conductive fabric, comprising an electrode body, a copper plate, and an electrode shell, as Figure 1b illustrates. The electrode body was fabricated with a conductive fabric enveloped in an elastic foam, as Figure 1b depicts. This configuration allows the conductive fabric, supported by the foam, to conform to the irregularities on the scalp surface and hairy regions. When the electrode was pressed to the scalp, its remarkable geometric consistency enhanced the contact area, reducing the electrode–skin contact impedance [26]. Figure 1c presents the design of the electrode shell model, alongside a 3D-printed prototype. The base of the electrode shell is equipped with a chamber designed to accommodate both the copper plate and the electrode body. A central groove is incorporated within the shell, facilitating the passage of wiring that connects to the copper plate. Additionally, a pair of clamp pins featuring apertures at the top are engineered to stabilize the electrodes during EEG signal acquisition. A copper plate serves as an adhesion layer to effectively connect the electrode body to the wiring, thereby enabling signal transmission. Figure 1d depicts the constructed fabric dry electrode. The dimensions of the electrode body are specified as 10 mm (L) × 8 mm (W) × 4 mm (H), ensuring that the fabric dry electrode has an effective contact area of 80 mm^2^, which aligns with the standard Ag/AgCl wet electrodes (OpenBCI, https://shop.openbci.com accessed on 21 March 2024).

### 2.5. PEDOT: PSS/PVA Fabric Dry Electrode Characterization

#### 2.5.1. Measurement of Impedance

The contact impedance at the interface between the electrode and skin for the PEDOT: PSS/PVA fabric dry electrode was systematically evaluated using the OpenBCI Cyton Board following the experimental setup described in Section 3.2. An Ag/AgCl wet electrode (OpenBCI, https://shop.openbci.com accessed on 3 April 2024) and a finger dry electrode developed by Florida Research Instrument Inc., Cocoa Beach, FL, USA, were also utilized for comparative analysis. All electrodes were positioned on the forehead, maintaining a separation of 1 cm between each pair of electrodes [27]. The contact impedance was recorded at 5 min intervals for 60 min, employing the Cyton Signal Widget in Impedance Mode (GUI Widget Guide | OpenBCI Documentation).

#### 2.5.2. Measurement of Short Circuit Noise

The short circuit noise of the PEDOT: PSS/PVA fabric dry electrode, the Ag/AgCl wet electrode, and the finger dry electrode were measured by acquiring the signal from electrodes attached to a polished silver plate (10 cm × 10 cm) [8]. The fabric dry electrode was tested first. One electrode was affixed to the silver plate, followed by the placement of another electrode connected to the EEG recording channel adjacent to it; finally, the reference electrode was secured next to the recording electrode on the plate. After a 60 s noise recording, all fabric dry electrodes were replaced with the Ag/AgCl wet electrodes, and then the finger dry electrodes were measured for a 60 s noise recording. The acquired noise was subsequently analyzed in the time domain.

#### 2.5.3. Measurement of EEG Signals

**Figure 2:**
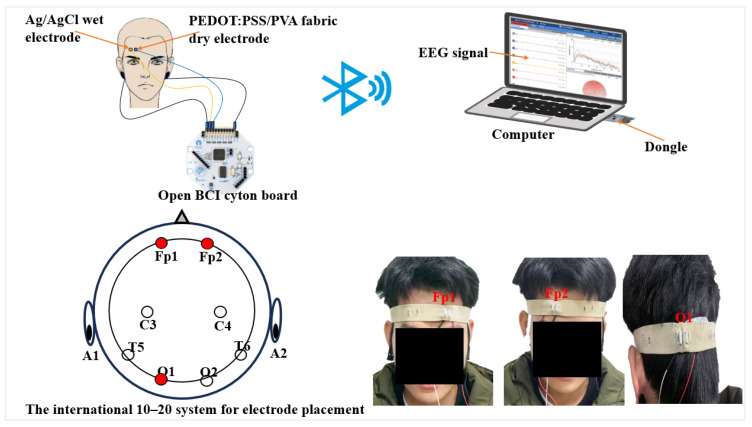
The schematic illustration of the setup for measuring EEG signals.

To assess the efficacy of the PEDOT: PSS/PVA fabric dry electrode in collecting EEG signals, a comparative analysis was conducted against the Ag/AgCl wet electrode. Both electrode types were interfaced with the OpenBCI Cyton Board for EEG signal acquisition following the setup depicted in Figure 2. The recorded signals were subsequently processed and visualized through the OpenBCI GUI on a laptop. The sampling frequency was configured at 250 Hz. Electrode placement adhered to the International 10–20 Electrode System [28,29]. Moreover, one reference electrode and one ground electrode were positioned on the left and right ears (A1, A2), respectively. An elastic bandage was employed to secure the electrodes in place.

Two electrodes were strategically positioned on the forehead (Fp1, Fp2) to capture EEG signals in a resting state for 30 s, followed by a 30 s EEG artifact measurement. During the EEG artifact measurement, participants were instructed to blink or clench for 1 s every 5 s. The Ag/AgCl wet electrode was placed 1 cm apart from the PEDOT: PSS/PVA fabric dry electrode. To facilitate the measurement of alpha waves, an additional electrode was affixed to the occipital lobe (O1), which is located within hairy regions of the scalp. Participants were required to close their eyes for 15 s and subsequently open their eyes for another 15 s to both stimulate and restrict alpha rhythm activity. Five subjects participated in the study, comprising two males and three females.

#### 2.5.4. Measurement of Signal-to-Noise Ratios (SNRs)

To conduct a more quantitative evaluation of the quality of the EEG signals, the signal-to-noise ratios (SNRs) were computed based on the spectral responses (SRs) using Equation (2). The methodologies employed for EEG signal processing and analysis were consistent with the findings presented in studies [30,31].

(2) $$\mathrm{SNR}\;(\mathrm{dB})=10\;\times \;\log_{10}\frac{signal\;power}{noise\;power}$$

#### 2.5.5. Measurement of Sweat Resistance

To explore the impact of prolonged use on electrodes which are exposed to sweat, we conducted a sweat resistance test on the fabric dry electrode following ISO-3160-2015 [32]. An artificial sweat solution mixed with 20 g/L NaCl, 17.5 g/L NH_4_Cl, 5 g/L CH_4_N_2_O, 2.5 g/L CH_3_COOH, 15 g/L C_3_H_6_O_3_, and 80 g/L NaOH was first prepared with a pH of 4.7. This solution was sprayed onto the electrode surface to mimic human sweating states: insensible perspiration with a spray volume of 0.28 g and sensible perspiration with a spray volume of 0.56 g. Subsequently, EEG signals were recorded using processed electrodes at Fp1.

#### 2.5.6. Measurement of Biocompatibility

The biocompatibility of the fabric dry electrode was assessed according to ISO10993-10-2021 [33]. The electrode was attached to the forehead using an elastic bandage for 4 h, and the skin condition was evaluated after it was removed.

## 3. Results and Discussion

### 3.1. Conductive Fabric Properties Analysis

The thickness measurement showed an increase of 26.67%, rising from 0.15 mm to 0.19 mm. This significant change in thickness indicates that a thick coating exists on the fabric surface. It is essential to verify whether the textile properties of the fabric have been impacted. Additionally, in the preparation of EEG electrodes the conductive fabric must undergo stretching and folding processes, which necessitates an analysis of its tensile properties. Notably, the tensile strength at break for the conductive fabric exhibited an increase from 266.92 N to 326.65 N, representing a significant improvement of 22.38% in the warp direction, and from 401.50 N to 441.94 N, indicating a 10.07% enhancement in the weft direction. This enhancement in tensile strength is likely attributable to the penetration of conductive paste into the interstitial spaces among the fibers and yarns, thereby augmenting friction and enhancing the overall breaking strength of the fabric. Furthermore, an increase in elongation at break was observed, with a rise of 6.15% in the warp direction and 5.78% in the weft direction. This phenomenon may be ascribed to the presence of PVA on the fiber surfaces. PVA possesses a flexible molecular structure that facilitates enhanced mobility during stretching. The synergistic effect of PVA contributes to the increased elongation at break [34]. It is suggested that the conductive fabric maintains its inherent textile properties, and the enhancement of its tensile properties is advantageous for both durability and fastness.

**Figure 3:**
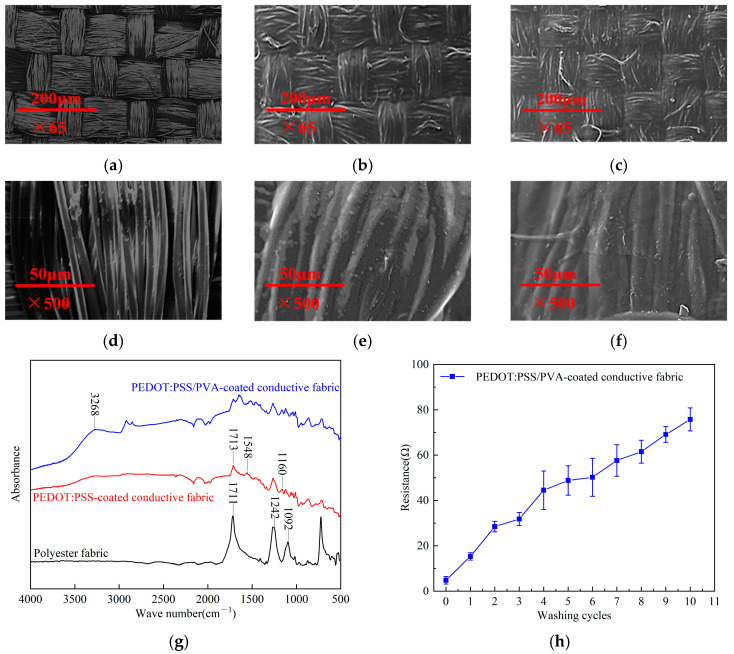
(**a**) SEM image of the polyester fabric (×65); (**b**) SEM image of the polyester fabric (×500); (**c**) SEM image of PEDOT: PSS/PVA-coated conductive fabric (×65); (**d**) SEM image of PEDOT: PSS/PVA-coated conductive fabric (×500); (**e**) SEM image of PEDOT: PSS/PVA-coated conductive fabric after 10 washing cycles (×65); (**f**) SEM image of PEDOT: PSS/PVA-coated conductive fabric after 10 washing cycles (×500); (**g**) FTIR spectra of the polyester fabric, PEDOT: PSS-coated conductive fabric, and PEDOT: PSS/PVA-coated conductive fabric; (**h**) The resistance of PEDOT: PSS/PVA-coated conductive fabrics after various washing cycles.

An investigation was conducted to assess the adhesion properties of conductive coatings on fabrics through surface morphology analysis utilizing SEM. Figure 3a,b,d,e present the SEM images depicting both untreated polyester fabric and the PEDOT: PSS/PVA-coated conductive fabric. The findings revealed a uniformly applied conductive coating on the fabric, with the conductive polymer composite filling the yarn interstices. During the synthesis of PEDOT: PSS, a small number of unreacted EDOT monomers may remain, potentially existing in the PEDOT: PSS system either in free form or as oligomers. The minimal presence of EDOT particles on the yarn surface indicates the favorable miscibility of PEDOT: PSS and PVA, forming a compact, homogeneous film over the fabric that constructs a continuous and complete conductive network.

Afterwards, the surface resistance of the coated conductive fabric, measured at four distinct positions, was 4.76 Ω, 5.02 Ω, 4.94 Ω, and 4.64 Ω, respectively. The average resistivity was calculated to be 0.09 Ω·cm. The average surface resistance, along with the standard deviation and margin of error, was 4.84 ± 0.07 Ω (1.5%), within a 95% confidence interval (CI). This further demonstrates that the coated conductive fabric has good conductivity and coating uniformity.

Figure 3g illustrates the FTIR spectra of untreated polyester fabric, PEDOT: PSS-coated conductive fabric, and PEDOT: PSS/PVA-coated conductive fabric. The FTIR spectra of the untreated polyester fabric revealed several characteristic absorption peaks: C=O at 1711 cm^−1^, C-O at 1242 cm^−1^, and -CH₂ at 1092 cm^−1^ stretching vibrations [35]. In the spectrum of PEDOT: PSS-coated conductive fabric devoid of PVA, the peak at 1713 cm^−1^ was attributed to the C=C stretching vibration of the aromatic ring of PSS, while the 1548 cm^−1^ peak was associated with the C=C stretching vibration of the thiophene ring present in PEDOT; additionally, the peak at 1160 cm^−1^ corresponded to the absorption peak indicative of the disulfonic acid group in PSS [36]. In contrast, the spectrum of PEDOT: PSS/PVA-coated conductive fabric demonstrated a prominent O-H stretching vibration near 3286 cm^−1^, accompanied by a red shift phenomenon [37]. Notably, no O-H stretching vibration was observed in the spectra of either the untreated polyester or the PEDOT: PSS-coated conductive fabric. This can be attributed to the interaction between the -OH (hydroxyl groups) of PVA and the oxygen-containing functional groups of PEDOT: PSS [38], thereby confirming the presence of PVA. Collectively, the FTIR analysis substantiates the presence of both PEDOT: PSS and PVA within the fabric matrix.

Moreover, the PEDOT: PSS/PVA-coated conductive fabric demonstrated stability over 10 washing cycles. As illustrated in Figure 3c,f, SEM images of the coated fabric after washing reveal that the conductive coating remains intact, with few protruding yarns following the washes. Furthermore, Figure 3h presents the resistance measurements of the PEDOT: PSS/PVA-coated conductive fabric after multiple washing cycles. The resistivity of the fabric after 10 washing cycles was determined at 1.35 Ω·cm. Although a decrease in resistance was observed, the fabric continued to exhibit commendable conductivity. This favorable washability can be attributed to incorporating PVA as a fixing agent.

### 3.2. Electrode–Skin Contact Impedance Analysis

**Figure 4:**
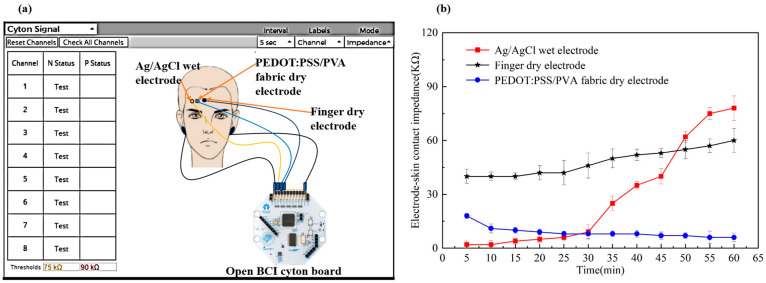
(**a**) Schematic illustration of the setup for measuring electrode–skin contact impedance using the OpenBCI Cyton Board; (**b**) The electrode–skin contact impedances of EEG electrodes over time, including Ag/AgCl wet electrode, finger dry electrode, and PEDOT: PSS/PVA fabric dry electrode.

Figure 4b illustrates a notable trend in the electrode–skin contact impedance across different types of electrodes over time. The contact impedance of the Ag/AgCl wet electrode exhibited a slight increase during the first 30 min; it surpassed that of the PEDOT: PSS/PVA fabric dry electrode at the 30 min mark and continued to increase sharply thereafter, ultimately exceeding the impedance of the finger dry electrode. This phenomenon may be attributed to the dehydration and consequent hardening of the conductive gel, which leads to a marked elevation in the contact impedance of the Ag/AgCl wet electrode [39]. Conversely, the contact impedance of the finger dry electrode showed minimal fluctuations during the initial 30 min, with a gradual increase observable after 30 min while maintaining the highest impedance levels. In contrast, the PEDOT: PSS/PVA fabric dry electrode remained relatively stable, gradually decreasing impedance over time. This stability can be explained by the high geometric consistency of the fabric dry electrode, which is wrapped in foam, thereby ensuring consistent skin contact that promotes low and stable contact impedance. Furthermore, the fabric dry electrode could absorb sweat and take it as an electrolyte, reducing contact impedance, an effect not observed in the finger dry electrode coated with metal [20].

The one-way ANOVA with a 95% CI indicated a statistically significant difference in contact impedance between the PEDOT: PSS/PVA fabric dry electrode and the Ag/AgCl wet electrode after 30 min (*p* < 0.05). Additionally, one-way ANOVA revealed no significant difference in the impedance of the PEDOT: PSS/PVA fabric dry electrode when comparing the initial 30 min to the subsequent period (*p* > 0.05). This observation suggests a substantial uniformity in electrode–skin contact impedance for the fabric dry electrode. Consequently, the PEDOT: PSS/PVA fabric dry electrode demonstrates a potential advantage over the Ag/AgCl wet and finger dry electrodes for long-term EEG monitoring, particularly in wearable applications. Furthermore, the contact impedance of the fabric dry electrode ranged from 6 to 18 kΩ, which fulfills the requisite criteria for use as an electrode in EEG measurement [26,40].

### 3.3. Short Circuit Noise Analysis

**Figure 5:**
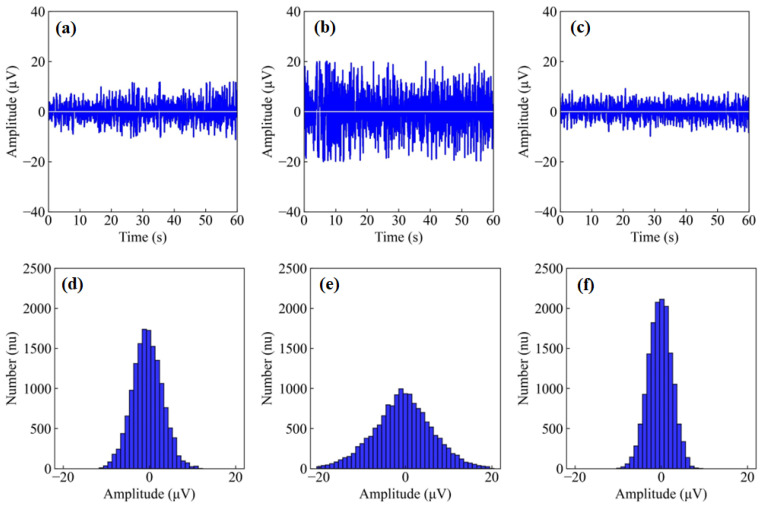
Results of the short circuit noise test: (**a**) noise measured with Ag/AgCl wet electrodes; (**b**) noise measured with finger dry electrodes; (**c**) noise measured with PEDOT: PSS/PVA fabric dry electrodes; (**d**) number of noises measured with Ag/AgCl wet electrodes at varying amplitudes; (**e**) number of noises measured with finger dry electrodes at varying amplitudes; (**f**) number of noises measured with PEDOT: PSS/PVA fabric dry electrodes at varying amplitudes.

Figure 5 shows the short circuit noise of the Ag/AgCl wet electrode, the finger dry electrode, and the PEDOT: PSS/PVA fabric dry electrode for 60 s. The findings indicate that the fabric dry electrode, as Figure 5c depicts, achieved the lowest and most stable noise level, which may be attributed to the superior flexibility and conductivity of the conductive fabric [41]. In contrast, the application of conductive gel appeared to increase the noise associated with the Ag/AgCl wet electrodes, as Figure 5a shows. The finger dry electrode, represented in Figure 5b, exhibited the highest noise levels, potentially due to the rigidity of the metal material employed. Additionally, Figure 5f demonstrates that the fabric dry electrode registered the least noise power, as the noise distribution is predominantly concentrated around 0 μV. Figure 5d,e present the number of noise signals across varying amplitudes recorded by the Ag/AgCl wet and finger dry electrodes, respectively. Furthermore, the short circuit noise follows a normal distribution characterized by random Gaussian noise, which can be effectively mitigated by applying various signal processing techniques, such as wavelet denoising [42]. These observations suggest that the fabric dry electrodes experience less interference compared to the wet and finger electrodes.

### 3.4. EEG Signals Analysis

**Figure 6:**
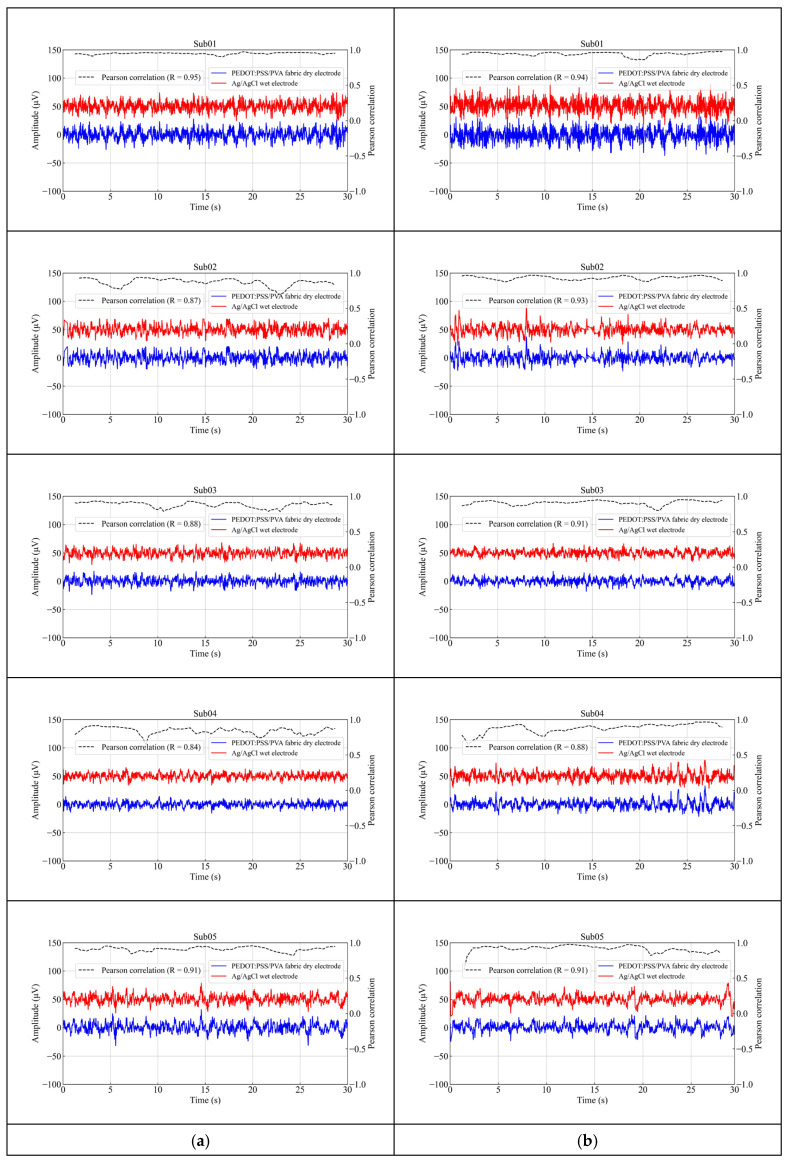
EEG signals were recorded for 30 s using the Ag/AgCl wet electrode and the PEDOT: PSS/PVA fabric dry electrode placed on the forehead of 5 subjects during a resting state. (**a**) Fp1; (**b**) Fp2.

Afterwards, a comparative analysis of the signal quality between the Ag/AgCl wet electrode and the PEDOT: PSS/PVA fabric dry electrode was conducted. Figure 6 illustrates a section of 30 s EEG recordings from five subjects collected from the forehead (Fp1 and Fp2) during a resting state. The findings indicated that the amplitude of EEG signals collected by the fabric dry electrode exhibited consistency with those recorded by the wet electrode, thereby suggesting that the fabric dry electrode could accurately acquire EEG signals. The correlation coefficient has been widely used to assess the degree of linear correlation between two datasets. In this study, the average correlation coefficient between the EEG signals recorded by the wet electrode and the fabric dry electrode at the forehead was determined to be 0.90 (0.89 at Fp1 and 0.91 at Fp2). These results demonstrated that the signal quality obtained from the fabric dry electrode was comparable to that of the wet electrode. This may be attributed to the ion–electron conductivity of PEDOT: PSS [19], which functions similarly to the conductive gel employed in wet electrodes, thereby effectively facilitating the acquisition of biopotentials.

**Figure 7:**
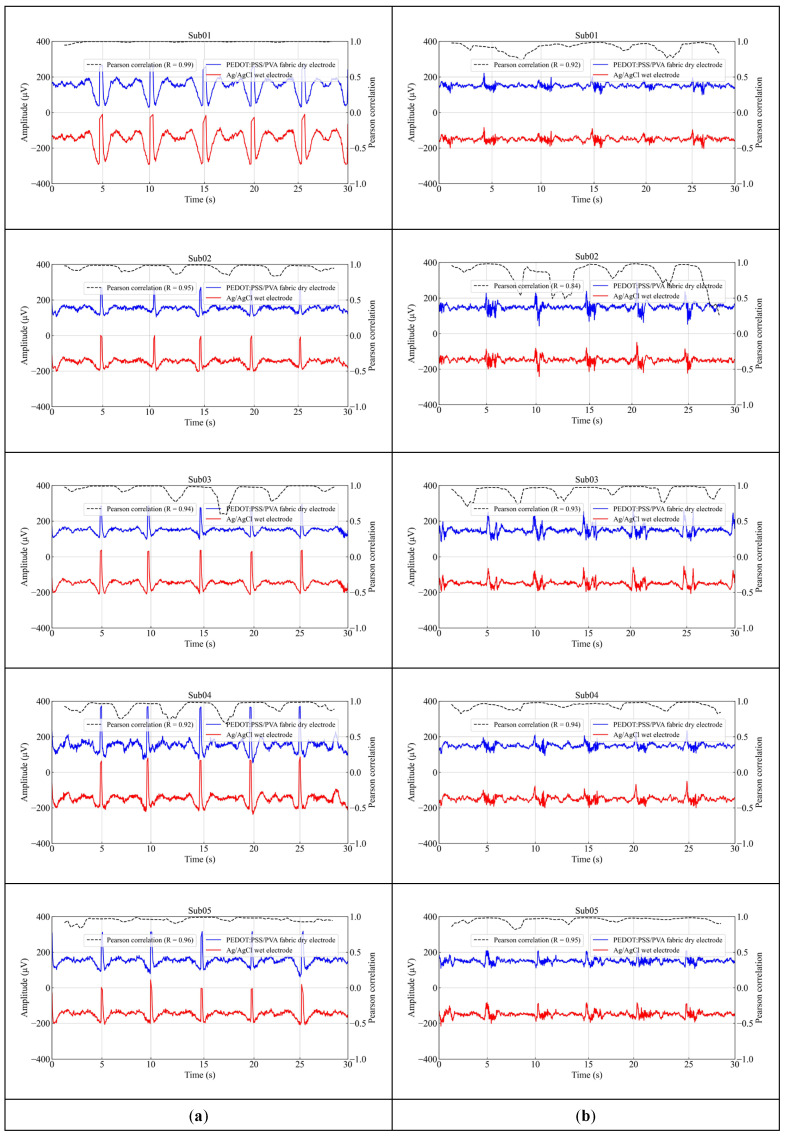
EEG artifacts were recorded for 30 s using the Ag/AgCl wet electrode and the PEDOT: PSS/PVA fabric dry electrode at Fp1 from 5 subjects. (**a**) Eye blinks; (**b**) teeth clenching.

In addition, the EEG artifacts recorded using the Ag/AgCl wet electrodes and the PEDOT: PSS/PVA fabric dry electrode were investigated. As Figure 7 shows, eye blinks and teeth clenching were consistently observed from both electrode types, aligning with the instructions provided to the subjects. For eye blinks and teeth clenching measurements, the average correlation coefficients between the Ag/AgCl wet electrode and the fabric dry electrode were 0.95 and 0.92, respectively. These findings suggest that the signal quality obtained with the proposed fabric dry electrode is nearly equivalent to that of the wet electrode.

**Figure 8:**
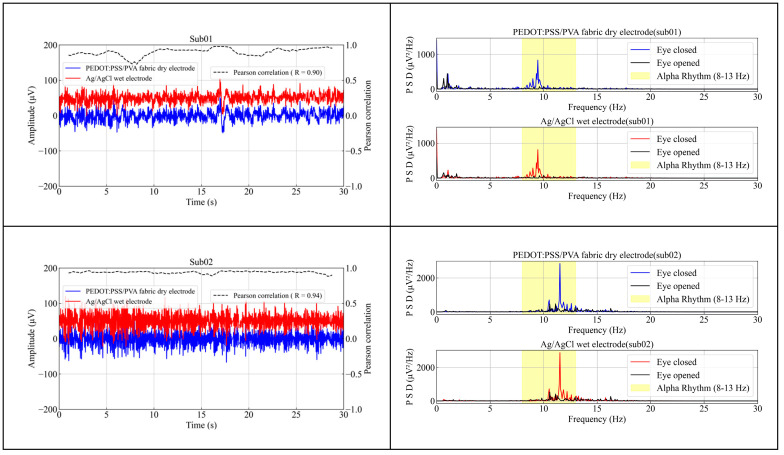

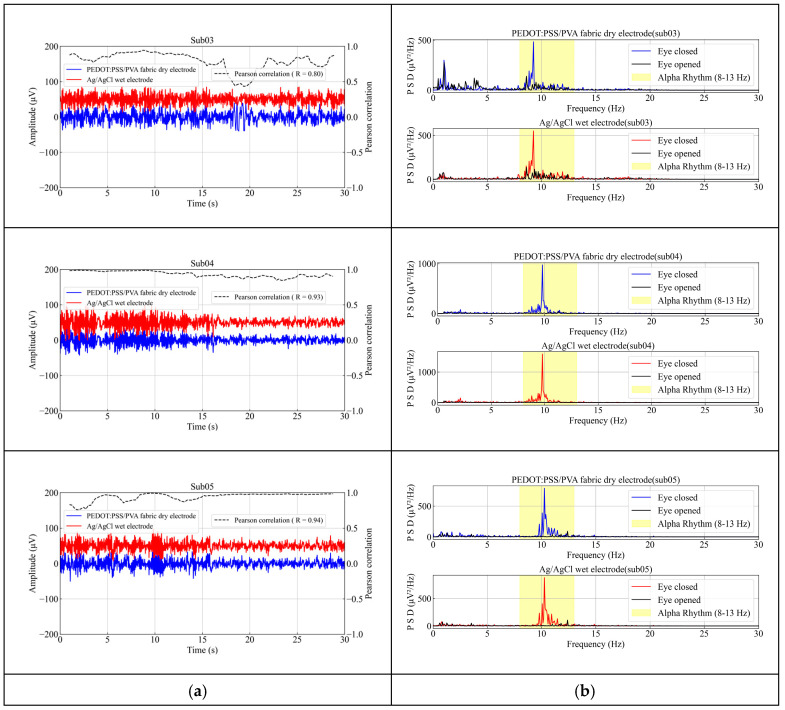
Results of EEG signal recordings for 30 s using the Ag/Ag/Cl wet electrode and the PEDOT: PSS/PVA fabric dry electrode from hairy areas (O1) from 5 subjects with the eyes closed/opened; (**a**) EEG signals; (**b**) PSD of EEG signals.

Furthermore, the alpha rhythms detected by the Ag/AgCl wet electrode and the PEDOT: PSS/PVA fabric dry electrode were examined in the study. Figure 8a presents the EEG signals recorded by both electrodes during the closing and opening of the eyes at hairy areas (O1). It is evident from Figure 8a that the first 15 s of EEG signals were recorded with the eyes closed, followed by another 15 s with the eyes opened. The amplitudes of the EEG signals with eyes closed were significantly higher than those with eyes opened [43]. These results indicated that both electrode types could discriminate well between the closed and open eye conditions [44]. Figure 8b displays the power spectral density (PSD) of EEG signals recorded by both electrode types. The distinct and similar alpha rhythms, ranging from 8 to 13 Hz, were observed from both electrode types when the eyes were closed, whereas the alpha rhythms were significantly restrained when the eyes were opened. Notably, the PSD of EEG signals measured by the fabric dry electrode demonstrated a similar tendency to that of the wet electrode. Consequently, it can be concluded that the fabric dry electrode and Ag/AgCl wet electrode exhibited comparable performance in alpha rhythms measurement.

Moreover, the average correlation coefficient between the EEG signals collected from the Ag/AgCl wet electrode and the PEDOT: PSS/PVA fabric dry electrode at hairy areas (O1) was determined to be 0.90, exceeding previously reported values [27]. This finding verified that the fabric dry electrode could accurately capture EEG signals from hairy areas, with signal quality closely approximating that of the Ag/AgCl wet electrode. Given its ease of installation, good user comfort, and high signal quality, the proposed fabric dry electrode could be considered a viable alternative to the traditional wet and metal dry electrode in brain–computer interface (BCI) applications, especially in real-life situations.

### 3.5. Signal-to-Noise Ratios (SNRs) Analysis

**Figure 9:**
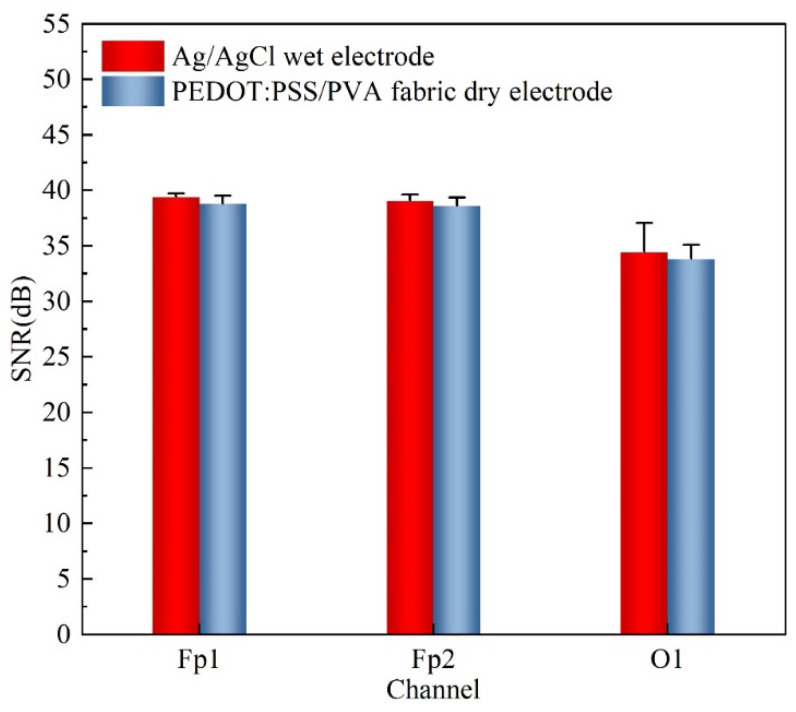
SNRs of the Ag/AgCl wet electrode and the PEDOT: PSS/PVA fabric dry electrode at Fp1, Fp2, and O1.

To quantitatively assess the specific qualities of EEG signals across different paradigms, SNRs were computed for the resting state (recorded at the forehead) and the eyes closed/open conditions (recorded at hairy sites). Figure 9 depicts the SNRs for all subjects, measured at the forehead (Fp1 and Fp2) and the hairy sites (O1). The obtained SNRs for the fabric electrode at Fp1, Fp2, and O1 were found to be 38.82 dB, 38.61 dB, and 34.46 dB, respectively. A comparative analysis of the SNRs between the Ag/Ag/Cl wet electrode and the PEDOT: PSS/PVA fabric dry electrode revealed that the SNRs of the fabric dry electrode were quantitatively analogous to those of the wet electrode.

### 3.6. Washability Analysis

**Figure 10:**
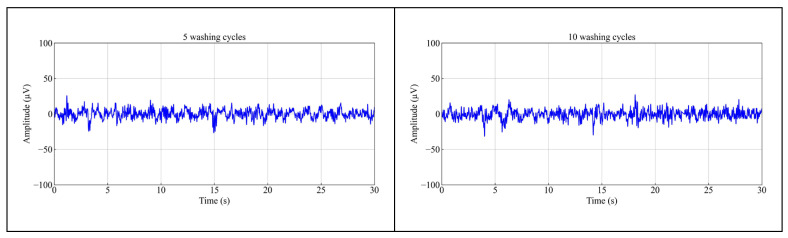
Results of EEG signal recordings for 30 s using the PEDOT: PSS/PVA fabric dry electrode after 5 and 10 washing cycles at Fp1.

Figure 10 illustrates the EEG signals obtained from the PEDOT: PSS/PVA fabric dry electrodes after five and ten washing cycles during a resting state (recorded at Fp1). It can be observed that the EEG signals recorded from the fabric dry electrode remain stable and consistent with those collected from the unwashed electrodes, even after 10 washing cycles. Although the amplitudes became smaller after washing, these fluctuations remain within an effective range (0.5~100 μv) [45]. Based on the one-way ANOVA at a 95% CI, it revealed no significant difference in the amplitudes of EEG signals between the unwashed electrodes and those subjected to 10 washing cycles, *p* > 0.05. Furthermore, the SNRs of the fabric dry electrode after 10 washing cycles were determined to be 37.67 dB, reflecting a minor decrease of −2.96%. The enhancement in washability can be attributed to the incorporation of PVA, which was purposely integrated during the coating for this purpose. The PEDOT: PSS/PVA conductive paste demonstrated strong adhesion to the polyester fabric due to the formation of numerous hydrogen bonds resulting from the interaction between the -OH of PVA and the -SO_3_H of PSS [22], thus ensuring the fastness of the conductive coating.

### 3.7. Sweat Resistance Analysis

**Figure 11:**
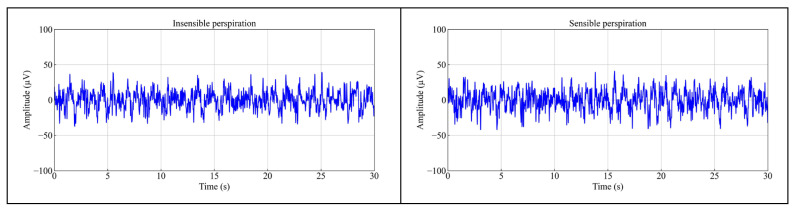
Result of EEG signal recordings for 30 s using the PEDOT: PSS/PVA fabric dry electrode simulating insensible perspiration and sensible perspiration at Fp1.

Figure 11 illustrates the EEG signals obtained from the PEDOT: PSS/PVA fabric dry electrodes that simulate insensible and sensible perspiration states at Fp1. The signals obtained from both perspiration states show the same amplitudes with the pristine fabric electrode, indicating no significant distortion or loss of signal quality. Based on the one-way ANOVA at a 95% CI, it revealed no significant difference in the amplitudes of EEG signals under both perspiration states, *p* > 0.05. Furthermore, the SNRs for insensible and sensible perspiration states are 38.69 dB (−0.33%) and 37.70 (−2.88%) dB, respectively. It is indicated that under insensible perspiration there is almost no change in SNRs; under sensible perspiration there is a very minor decrease in SNRs. It can be concluded that the fabric dry electrode has good sweat resistance.

### 3.8. Biocompatibility Analysis

**Figure 12:**
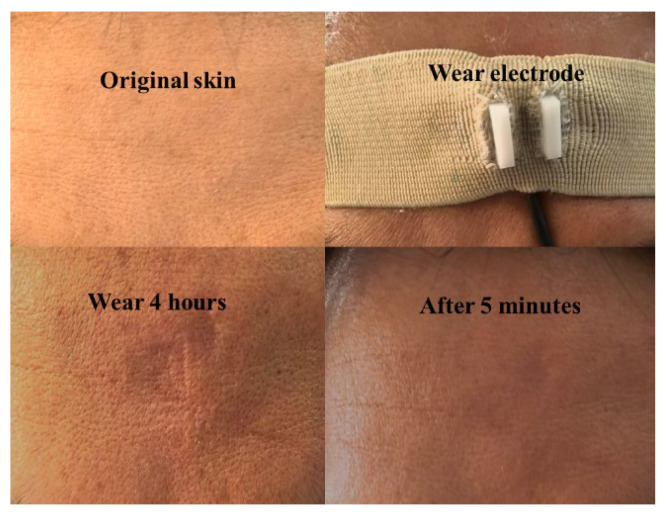
The skin condition test of wearing the PEDOT: PSS/PVA fabric dry electrode for 4 h.

Figure 12 illustrates the skin condition assessment following 4 h of wearing the PEDOT: PSS/PVA dry electrode fabric. During this period, the participants reported no pain or discomfort. While traces from the electrode imprints were noted on the skin, there were no indications of tissue injury or allergic reactions. Remarkably, these imprints disappeared completely within 5 min, with the skin reverting to its baseline appearance. Consequently, it can be concluded that the fabric dry electrode demonstrates exceptional biocompatibility and comfort for users during prolonged monitoring.

## 4. Conclusions

This research presents the successful development of an EEG dry electrode utilizing PEDOT: PSS/PVA-coated conductive fabric for brain activity measurement. This novel fabric dry electrode exhibits several noteworthy characteristics, such as the following: (1) it’s gel-free, facilitating ease of operation and enabling long-term EEG monitoring; (2) the design incorporates conductive fabric enveloped in foam which effectively adapts to the irregularities of the scalp surfaces, ensuring optimal skin contact and achieving low and stable contact impedance; (3) the electrode demonstrates excellent washability, sweat resistance, and biocompatibility; (4) it retains the fundamental properties of textiles, being both flexible and stretchable, facilitating user comfort. Results indicate that the contact impedance of the proposed fabric dry electrode was lower than that of the Ag/AgCl wet electrode after 30 min, with minimal short-circuit noise observed. Furthermore, the EEG signals acquired via the fabric dry electrodes closely resemble those obtained from the wet electrodes, even in hairy regions with high SNRs. Consequently, this electrode is anticipated to be a valuable tool for long-term EEG monitoring, particularly in wearable health monitoring applications.

Despite its contributions, the study has limitations. The electrode utilized conductive fabric, yet only its tensile properties were examined; other textile qualities, such as bending properties and breathability, require further study. The effect of bending and multiple uses on EEG signal quality should also be studied in the future. The factors influencing contact impedance, such as contact area and hair density, as well as their relationship, require further investigation. Moreover, EEG signal monitoring should be performed over an extended period for comprehensive long-term assessment.

## Data Availability

The data presented in this study are available on reasonable request from the corresponding author. The data are not publicly available due to privacy reasons.
